# Prevalence of heterophilic antibodies in serum samples from horses in an equine hospital, and elimination of interference using chicken IgY

**DOI:** 10.1186/s13028-021-00575-1

**Published:** 2021-03-12

**Authors:** Bo Dong, Daniel Bergman, Bodil Ström Holst

**Affiliations:** 1 Department of Clinical Sciences, Swedish University of Agricultural Sciences, Box 7054, 750 07 Uppsala, Sweden; 2 College of Life Science of Longyan University, Longyan, 364012 China

**Keywords:** ELISA, Heterophilic antibodies, Horse, Interference, Serum

## Abstract

**Background:**

Heterophilic antibodies in serum and plasma can interfere with mammalian antibodies in immunoassays and result in false test results, usually false positive. Although studies screening for heterophilic antibodies as well as elimination studies have been conducted in dogs and cats, knowledge of the presence of heterophilic antibodies in other species in veterinary medicine is limited. In this study, a 2-site sandwich-type interference assay that detects anti-mouse antibodies was used to detect heterophilic antibodies in a population of horses treated in an animal hospital.

**Results:**

A total of 194 serum samples from 127 individual horses were analyzed. There were 11/127 (8.7%) interference-positive horses, and these were analyzed in an assay exchanging the capture mouse IgG with chicken IgY. The positive samples were negative in the chicken IgY assay, indicating elimination of a possible interference, with the chicken-based assay. Four interference-positive samples were from geldings, and anti-Müllerian hormone (AMH) was analyzed from these samples. AMH concentrations were negative in these samples as expected in geldings, indicating that the heterophilic antibodies did not cause interference in the AMH assay.

**Conclusion:**

The present study shows that there are heterophilic antibodies in horse serum samples like in samples from humans, dogs, and cats. The use of chicken-based reagents, such as chicken IgY, which do not cross-react with mammalian IgG, eliminates the effects of interfering antibodies in the samples. Equine heterophilic antibodies do not necessarily cause interference in commercial immunoassays.

## Background

Immunoassays are commonly used in veterinary clinical practice, especially for hormone analyses, and provide support for clinical diagnosis and treatment. One assay that is commonly used is the sandwich immunoassay [1]. This assay has the advantage of being very sensitive, but it is also prone to interference by heterophilic antibodies [1]. Heterophilic antibodies can cross-link capture antibodies with detection antibodies and have been shown to cause false-positive results in human medicine [2–7], for anti-Müllerian hormone (AMH) and B-type natriuretic hormone in dogs [8, 9], and for equine growth hormone (eGH) in horses [10, 11]. In human medicine, heterophilic antibodies can be grouped as true heterophilic antibodies, human antimouse antibodies (HAMA) and rheumatoid factors (RF) [1, 12].

Reported prevalences of heterophilic antibodies vary and depend on methods used. A double-antibody sandwich immunoassay that does not cross-link with any known substance can be used to screen for heterophilic antibodies. In such an assay, signals may be generated by the cross-linking of the assay antibodies by heterophilic antibodies [1]. In veterinary medicine, an interference assay was used to study the prevalence of heterophilic antibodies in the serum of dogs and cats, and the prevalence was reported to be 5–9% [13]. In horses, it has been reported to be 5% [11]. In humans, it has been reported to be as high as 40% [14]. The frequency of interference in human serum samples has been reported to be from 0.5 to 2% to around 4% [15, 16]. It will vary with the assay used but will be lower than the prevalence of heterophilic antibodies [8, 16].

Most previous reports on screening and elimination of interfering antibodies in veterinary clinical laboratories have focused on dogs and cats [9, 13, 15]. In the horse, abnormally high concentrations of eGH analyzed using an in-house enzyme-linked immunosorbent assay (ELISA) have been described to be caused by heterophilic antibodies, and a screening revealed a presence of heterophilic antibodies in 5% of serum samples from healthy horses [10, 11].

Heterophilic antibodies are a heterogeneous group, and multiple strategies are required to eliminate their effect on assay results [17]. One approach is taking advantage of the fact that heterophilic antibodies against mammalian IgG do not cross-react with chicken IgY. The exchange of mouse IgG with chicken IgY has therefore been shown to eliminate the interference of heterophilic antibodies in human samples as well as in samples from dogs and cats [13, 18].

The goals of this study were to use a previously developed species-independent interference assay to screen a population of horses treated in animal hospitals for presence of heterophilic antibodies, to assess whether chicken IgY-based tests eliminate interference and if detected heterophilic antibodies cause interference in a commercial sandwich immunoassay for analysis of AMH.

## Methods

### Animals

Equine serum that had been analyzed at the Clinical Pathology Laboratory, the University Animal Hospital in Uppsala, Swedish University of Agricultural Sciences, Sweden was used. Exclusion criteria were clearly visible signs of hemolysis or lipemia.

### Interference assay

An interference assay was performed as described by Bergman and co-workers [13]. Negative samples from trial runs were pooled and used as negative controls. The chicken anti-mouse IgG was diluted 1:1600 in the pooled negative control sera and this solution represented the positive control. As standard, chicken anti-mouse IgG (Immunsystem AB, Uppsala, Sweden) was used, diluted in phosphate-buffered saline (PBS) 1:1600. The cutoff point was determined by calculating the mean optical density (OD) of the duplicates of a serial dilution seven times of the standard. For each run, the cutoff point was required to be greater than the assay limit of detection (LoD), as determined by the formula LoD = 0-standard + 2 standard deviations (SD) (doing 25 repeat measurements of the 0-standard). The intra-assay coefficients of variation (CVs) for the cutoff point ranged from 0.88 to 6.81%, and the inter-assay CV was 22.1%. A relative OD value for each sample was calculated by dividing the mean OD of the sample by the cutoff value. The cutoff limit for a positive result was > 1. Similarly, the positive control was required to be > 1 and the negative control < 1.

In brief, 100 µL purified mouse IgG (2 µg/mL) was added to each well of a polystyrene microtiter plate and placed overnight at 4 ºC. After washing with PBS, a total volume of 50 µL of standards, controls and samples were pipetted in duplicate into the wells followed by incubation at room temperature (RT). After another washing, monoclonal horseradish peroxidase (HRP)-conjugated mouse anti-human carcinoembryonic antigen (CEA) IgG was added, selected not to match the specificity of the capture antibody, thereby excluding the possibility that any existing analyte would give rise to a positive result. The plate was then incubated and washed. Finally, 3,3ʹ,5,5ʹ tetramethylbenzidinen (TMB) was added and the plate was incubated for 8 min at RT in the dark before adding stop solution (H_2_SO_4_) and reading OD at 450 nm.

### Interference elimination

Samples that were positive in the interference assay were further tested in a chicken-based assay, as previously described [13]. In brief, every second column of a microtiter plate was coated with either 2 µg/mL of purified mouse IgG or 2 µg/mL nonimmunized chicken IgY (Immunsystem AB). 50 µL of standards, controls and samples were added into each well, and incubated at RT. After washing, 100 µL of a 1:10,000 dilution of monoclonal HRP-conjugated mouse anti-human CEA IgG was added, followed by incubation at RT. The plate was washed, TMB was added and the plate was incubated at RT in the dark before adding stop solution and reading OD at 450 nm.

### Anti-Müllerian hormone assay

Samples from geldings that were positive in the interference assay were analyzed for AMH using an ELISA (AMH Gen II, Beckman coulter), according to the manufacturer. Briefly, 30 µL of standards, controls and samples were mixed with 150 µL assay buffer, and 120 µL of the so achieved solution were pipetted in duplicate into each well and incubated in an anti-AMH antibody coated microtitration plate. After incubation and washing, anti-AMH biotin conjugate was added, and after a second incubation and washing step, streptavidin-HRP was added. After a third incubation and washing step, the substrate, TMB, was added and incubated briefly before adding an acidic stopping solution. The degree of enzymatic turnover of the substrate was determined by dual wavelength absorbance measurement at 450 nm and 620 nm. The intra-assay CV was < 5% and the inter-assay CV was < 15%.

### Statistical analysis

Routine descriptive statistical methods were used. The effects of sex, breed, castration status and diagnosis were compared between the positive and the negative group using Student’s *t*-test. In the study of interference elimination, the Wilcoxon signed-rank test was used to compare results using mouse IgG with those using chicken IgY. The level of statistical significance was set at P < 0.05. The GraphPad Prism Software version 8.0 for Windows (San Diego, CA, USA) was used for statistical analyses.

## Results

### Horses

A total of 194 samples from 127 individual horses were analyzed. The horses were of 27 different breeds. Their median age was 8 years, interquartile range (IQR) 0.5–13.0 years, and included 59 mares, 22 stallions and 46 geldings. Forty-four horses were subjected to multiple analyses: 27 horses had samples analyzed twice, 10 horses three times, 5 four times and two horses had samples analyzed 5 times. All multiple samples were collected at different times. The horses were classified into 9 different categories according to diagnosis or cause for initial sampling (Table 1).

**Table 1:** Characterization of the horses screened for interfering antibodies by disease category

Disease category	Positive samples (n = 11)	%	Negative samples (n = 116)	%	Total (n = 127)
Bone, muscle and joint	4	21.1	15	78.9	19
Digestive tract	2	3.9	49	96.1	51
Infection/inflammation	2	9.5	19	90.5	21
Neoplastic disease	1	16.7	5	83.3	6
Neurologic disease	0	0	4	100	4
Reproductive disease	0	0	5	100	5
Respiratory disease	0	0	3	100	3
Skin disease	1	10	9	90	10
Other	1	16.7	5	83.3	6
SUM	11	8.7	116	91.3	127

### Interfering antibodies in horses

**Fig. 1:**
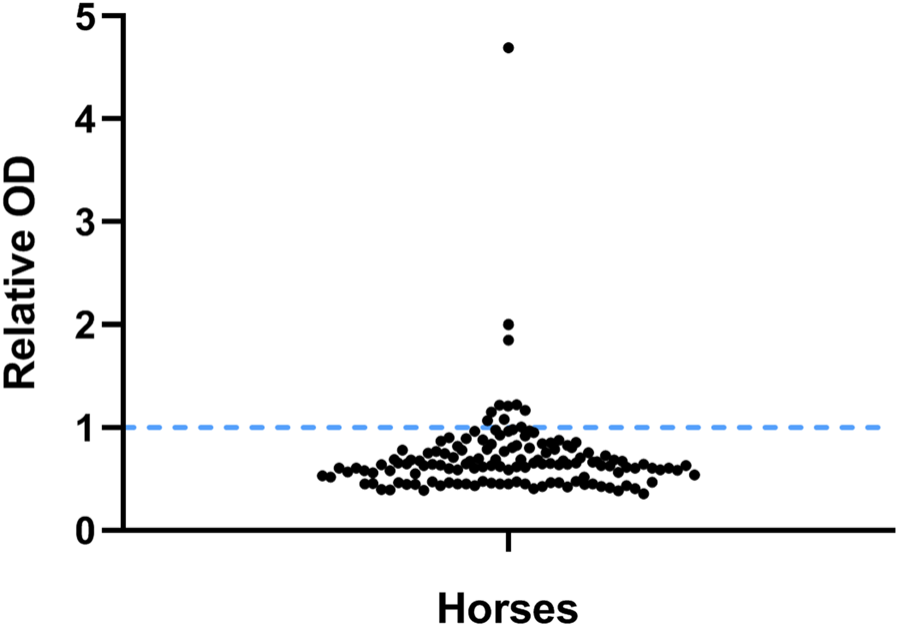
Screening results of interference assay. Horse samples (n = 127) were screened with a sandwich ELISA using nonimmunized mouse IgG as the capture antibody. Cutoff level is indicated by the dashed horizontal line

There were 11/127 (8.7%) interference-positive horses, with a median age of 12.0 years, range 1 month–17 years, (IQR 4.0–15.0 years), seven mares and four geldings of seven different breeds (Fig. 1).

The relative OD for the positive samples ranged between 1.01 and 4.69. A 15-year-old Icelandic horse gelding admitted to the animal hospital for liver disease was sampled three times within two weeks and was only positive for interference the second time (relative OD 1.2). A 14-year-old intact female Icelandic horse with a subcutaneous skin abscess was positive for interference the first sample (relative OD 1.03), but was negative on three subsequent samplings within three weeks. There was no significant difference of age, sex, breed, neutering status, or diagnostic category between interference-positive and interference-negative horses (P > 0.05).

### Interference elimination

**Fig. 2:**
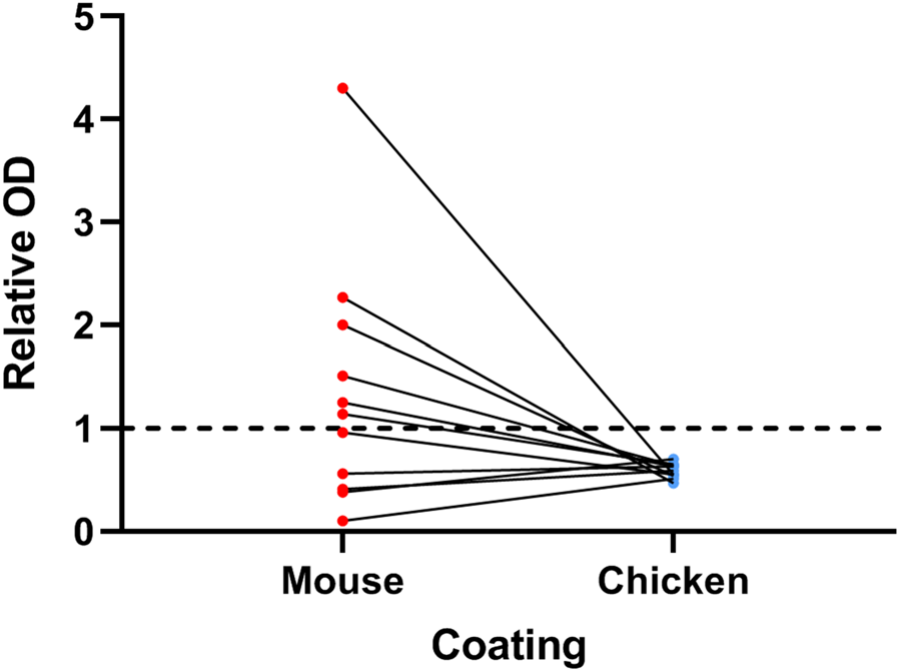
Results of interference elimination. The reactivity of heterophilic antibodies towards unimmunized mouse IgG and chicken IgY was detected with HRP- conjugated mouse anti-CEA antibody. The positive cutoff level is indicated by a dotted horizontal line

When chicken IgY antibody was used as the capture antibody, all the 11 interference-positive samples were negative. When mouse IgG was used, five samples were also detected negative, although they had previously been evaluated positive in screening tests (Fig. 2). The 11 samples produced significantly less signal in the wells containing chicken IgY than in wells containing mouse IgG (Z = − 3.059, P < 0.01).

### Analysis of anti-Müllerian hormone

Enough serum for AMH analysis was available from four geldings (relative OD 1.1; 1.2; 1.2 and 1.8), all of which had AMH concentrations below the lowest standard point.

## Discussion

In the present study, equine serum samples were screened for heterophilic antibodies and the prevalence was found to be 8.7% (11/127). This is similar but slightly higher than previously reported for horses [11], and similar to what has been reported for cats and dogs, using the same species-independent ELISA [13]. The horse population studied by Borromeo and co-workers [11] was healthy horses. The samples included in the present study were from horses admitted to an equine hospital, and the results may therefore better mirror the situation in clinical practice. Variations in prevalence of heterophilic antibodies may also be related to the methods used.

In human medicine, it has been shown that interference in immunoassays is a present and underestimated problem [16, 19]. The result achieved when affected by heterophilic antibodies is usually falsely increased, resulting in overestimation of the analytes in question. Examples of analyses that may be affected in equine medicine are e.g. adrenocorticotrophic hormone (ACTH) and insulin, used in the diagnosis of the two most common endocrine syndromes: pituitary pars intermedia dysfunction (PPID) and equine metabolic syndrome (EMS) [20], and erythropoietin, used for doping [21]. If the test results are clearly above the normal range, interference by heterophilic antibodies may be suspected, as was the case of interference with the in-house eGH ELISA [10]. In other cases, a result that is false high but within the normal range may not raise such a suspicion but lead to an incorrect diagnosis. Evaluating the effect of heterophilic antibodies on immunoassays is challenging, as the true result most often is unknown. Reanalyzing the sample after precipitation with polyethylene glycol (PEG) can result in normalized values in humans [22], but the method does not work well for samples from dogs [8]. One assay with a known expected result for castrated animals is the AMH immunoassay. In horses, this assay can be used to differ geldings, having non-detectable AMH concentrations, from cryptorchid stallions, having high AMH concentrations [23]. It is a sandwich immunoassay using murine antibodies, and thus similar to the interference assay. In the present study, AMH was analyzed in samples from four geldings with heterophilic antibodies. All samples had AMH concentrations below the detection limit, thus no sign of interference was detected. One possible reason for the lack of interference, leading to false positive results, is that the concentrations of heterophilic antibodies were just above the cut-off value, indicating a weak reactivity. Since antibodies affect immunoassay results both by their concentration and their affinity, it is also possible that the affinity of the heterophilic antibodies was too low to cause interference in the AMH assay [1]. In dogs, 2/7 samples with heterophilic antibodies caused a false positive result in the AMH assay, and these were the samples with the strongest anti-mouse reactivity. As commercial assays generally are designed to minimize the effect of heterophilic antibodies, the problem with interference is expected to increase with stronger reactivity.

That the positive results in the interference assay were caused by heterophilic antibodies is supported by the fact that they were eliminated with chicken IgY as capture antibodies, and that samples with visible signs of hemolysis and lipemia were excluded. In addition, all samples were cryopreserved at − 20 ºC to eliminate interference by complement [24]. Clinically relevant interference has previously been described with an in-house ELISA for eGH using mouse and rabbit antibodies [11]. The lack of interference with chicken IgY suggests that exchanging mammalian IgG with chicken IgY in immunoassays may reduce the risk of interference by heterophilic antibodies, as has previously been described [18].

Iatrogenic interference has been reported in human medicine as a result of monoclonal antibody therapy [25], but as no therapeutic antibodies are licensed for use in horses this is not a possible cause for the heterophilic antibodies in the present study. Direct physical contact with other species and indirect exposure through vaccination and food intake has also been suggested to potentially induce heterophilic antibodies [16]. Rheumatoid factors are considered as one of the possible causes of the interference, with age, gender and autoimmunity being main risk factors for interference caused by RF in human medicine [15, 26, 27]. In horses, there have been reports of an age-dependent increase in IgM RF regardless of sex [28]. Anamnestic data from horses with positive samples were reviewed to identify possible risk factors for interference. However, we found no significant relationship between the presence of heterophilic antibodies and age, sex, breed, castration status, or diagnosis.

Of the 11 positive horses, three were sampled and assayed on multiple occasions and tested positive only once, with less than a month between the different samples. A transient character and low affinity of heterophilic antibodies has been described [29, 30], although they have been described to persist for several years in dogs [31]. The reactivity of these samples was rather weak, and a variation between different runs may contribute to the shifting results.

## Conclusions

The prevalence of heterophilic antibodies in a hospital population of horses was similar to that previously reported for dogs and cats. Exchanging mammalian IgG with chicken IgY can reduce the problem of interference in immunoassays for equine samples.

## Data Availability

All data generated or analyzed during this study are included in this published article.

## Abbreviations

ACTH: Adrenocorticotrophic hormone
AMH: Anti-Müllerian hormone
CEA: Carcinoembryonic antigen
CV: Coefficient of variation
eGH: Equine growth hormone
ELISA: Enzyme-linked immunosorbent assay
EMS: Equine metabolic syndrome
HAMA: Human antimouse antibodies and
HRP: Horseradish peroxidase
IgG: Immunoglobulin G
IgM: Immunoglobulin M
IgY: Immunoglobulin Y
IQR: Interquartile range
LoD: Limit of detection
OD: Optical density
PBS: Phosphate buffered saline
PEG: Polyethylene glycol
PPID: Pituitary pars intermedia dysfunction
RF: Rheumatoid factor
RT: Room temperature
TMB: 3,3′,5,5′-Tetramethylbenzidine
